# Prevalence of risk factors of non-communicable diseases in the Sultanate of Oman: STEPS survey 2017

**DOI:** 10.1371/journal.pone.0259239

**Published:** 2021-10-28

**Authors:** Adhra Al-Mawali, Sathish Kumar Jayapal, Magdi Morsi, Waleed Al-Shekaili, Avinash Daniel Pinto, Hilal Al-Kharusi, Ayaman Al-Harrasi, Zainab Al-Balushi, John Idikula

**Affiliations:** 1 Centre of Studies & Research, Ministry of Health, Muscat, Sultanate of Oman; 2 Strategic Research Program for Non-Communicable Diseases, The Research Council (TRC), Muscat, Sultanate of Oman; All India Institute of Medical Sciences Bhopal, INDIA

## Abstract

**Background:**

Non-communicable diseases (NCD) represent a major public health issue and currently cause 185.75 deaths per 100,000 population in Oman. Hence, there is a need for comprehensive, up-to-date and internationally comparable data on NCD risk factors in order to evaluate the effectiveness of ongoing public health policies and to develop further NCD prevention and control interventions. The aim of the study was to provide evidence-based, up-to-date, extensive, and reliable baseline data on the behavioural and biological risk factors of NCDs in the Sultanate of Oman.

**Methods:**

A cross‐sectional, prospective, observational community‐based survey designed to be nationally representative of the Sultanate of Oman was conducted based on the WHO STEPwise approach to Surveillance (STEPS). Multi-stage stratified random sampling according to geographical distribution selected a total of 9053 households (Omani nationals and non-Omani residents). Cluster sampling was used to randomly select equal clusters from each governorate. 823 households were randomly selected from the list of all households in all selected clusters from each governorate and one eligible adult selected from each household randomly accounting for 6582 consenting participants. The survey used demographic and behavioural information questionnaires along with physical and biochemical measurements among adults aged 18 years and above.

**Results:**

The prevalence of behavioural risk factors such as tobacco use was 9%, alcohol consumption was 2%, insufficient fruit or vegetable intake was 61%, and insufficient physical activity was 39%. The prevalence of biological risk factors such as overweight and obesity was 66%, raised blood pressure was 33%, raised blood glucose was 16%, and raised blood cholesterol was 36%. The prevalence of multiple risk factors was also determined and 95% of the population were found to have more than one risk factor. Three or more risk factors were found among 33% of population aged 18 years and above and 45% of the population aged 45 years and above.

**Conclusion:**

A high prevalence of various NCD risk factors was found which needs to be addressed through health promotion, education, and policy. The findings are important to support the formulation and implementation of NCD-related policies and action plans that improve health status and prevent mortality due to NCDs in Oman.

## Introduction

Non-Communicable Diseases (NCDs) represent a major public health and broader societal problem, causing around 42 million deaths worldwide every year leading to high costs in health expenditures, absenteeism and loss of years of productive life [1]. The 4 sub-groups of NCDs that account for over 80% of all premature NCD deaths are cardiovascular diseases, cancers, chronic respiratory diseases, and diabetes. Worldwide, NCDs deaths are projected to increase by 17% by 2025 [2] with an estimated cumulative loss of output of $47 trillion between 2011 and 2030 [3]. The World Health Organization (WHO) estimated that there will be 2.4 million deaths from NCDs in the high, middle and low-income countries of the WHO Eastern Mediterranean Region by 2025 [4]. It is therefore unsurprising that several NCD-related indicators had been included in the final list of Sustainable Development Goals (SDG) indicators adopted by the United Nations General Assembly in July 2017 [5].

In the Sultanate of Oman, NCDs cause 185.75 deaths per 100,000 population [1]. These premature deaths seriously affect average life expectancy and quality of life. In spite of recent progress in addressing NCD and their risk factors, the latest previous health survey [6] conducted in Oman (2008) indicated that a significant fraction (70%) of Omani adults still had an insufficient intake of fruits and vegetables, 40% were physically inactive, and one in seven Omani men use tobacco. Also, more than 40% of adult Omanis had raised blood pressure and 12.3% had raised blood glucose [6].

Oman is one of the several countries selected by WHO to receive integrated support for rapid progress in achieving nine global targets for prevention and control of NCDs. Limited and fragmented data are currently available on the prevalence of NCD risk factors from previous surveys conducted in the past in the Sultanate of Oman. Thus, the need arose for comprehensive, up-to-date, and internationally comparable data on NCD risk factors in order to evaluate the effectiveness of ongoing public health policies and to develop further NCD prevention and control interventions. Hence, this STEPS survey aimed to assess the current prevalence of behavioural and biological risk factors of NCD in Oman.

## Materials and methods

### Study setting

Situated in the extreme south-eastern corner of the Arabian Peninsula, the Sultanate of Oman has is divided into eleven governorates (regions). As of 2017, Oman had a total population of 4.56 million with a sex ratio of 102 males per 100 females for the Omani population, literacy rate of 96.1, and life expectancy of 77.9 at birth for the total population [7].

### Study design and sampling technique

A national prospective, observational, cross‐sectional community‐based survey was conducted from January 2017 to April 2017 using a representative sample population of the Sultanate of Oman based on the WHO STEPwise approach to Surveillance (STEPS) of NCD risk factors [8, 9]. The population surveyed adults aged 18 years or older, men and women, as well as nationals and non-Omani residents. Those younger than 18 years old, tourists, persons with cognitive impairment, institutionalised persons who indicated their usual place of residence was a military base, and persons from labour camps were excluded. A multi-stage cluster sampling strategy was adopted to select 9053 households from 550 clusters across Oman (823 households from 11 governorates/regions of Oman). The sample was drawn from the 2010 census block area (clusters) with stratification done based on two factors: governorate and nationality (Omani and Non-Omani). The sample size was calculated based on a WHO STEPS standard formula (S1 Appendix) taking into account design effect. Equal sample selection was done to obtain the desired precision and estimates for each governorate/region. One eligible participant aged 18 years or older in each household was randomly selected (by the Kish method) to take part in the survey. If the eligible household was not available at the time of the visit, the team scheduled another convenient timing to visit and do the interview. A non-response was marked for those that were unavailable after repeated attempts. In order to overcome sampling bias, sample weights were calculated and adjusted according to the primary and secondary sampling units. Further details on sampling can be found on the Oman NCD Risk Factors Survey Report [10].

### Data collection

Data collectors, mainly nurses and health educators, were recruited in each governorate (region). Overall, 66 data collectors and 11 field supervisors were responsible for data collection. Furthermore, 1 regional coordinator, 1 information technology technician, and 1 laboratory technician were also recruited in each governorate. Urine samples were measured by the laboratory technicians in the health centre laboratories. A one-week training program was conducted for data collectors with interactive lectures, practical demonstrations, hands-on training, role plays and mock interviews to impart the survey objectives, field work staff duties, and essential data collection skills.

The WHO NCD STEPS instrument (Version 3.1) [11] consisted of three steps. **Step 1** consisted of face-to-face interviews using an advanced standardized country-specific version of the STEPS questionnaire and locally-adapted show cards. The original survey questionnaire comprised of 16 modules, containing 139 questions overall which included 78 core, 16 expanded, and 45 country-specific questions. These included socio-demographic characteristics, key behavioural risk factors, lifestyles, and history of chronic diseases. Due to the scale of the survey, only main components of the questionnaire have been represented in this article. The data collection was conducted in two languages, namely Arabic and English. The tool was validated by a well-established method of translation from the original English version into Arabic by an expert panel as well as back translated, adapted to the local environment and needs, and tested in terms of wording and understanding, in order for the final version to be conceptually equivalent in both versions. **Step 2** consisted of physical measurements (weight, height, waist and hip circumference), and blood pressure to investigate biological risk factors such as raised blood pressure, and overweight and obesity. Weight was recorded using a portable digital weighing scale with light clothing on and without shoes, with a precision of 0.1 kg. Similarly, adult portable stadiometers were used to measure height (in centimetres up to 0.1 cm) after removing shoes, socks, slippers and any head gear. Blood pressure measurements were done thrice at three-minute intervals, and the mean of the second and third readings were taken according to the STEPS protocol. Calibrated measuring instruments were used throughout the survey for height (SECA^®^ 213 portable stadiometer), weight (SECA^®^ 813 digital floor scale), and blood pressure (Omron digital blood pressure device). **Step 3** consisted of biochemical markers levels (fasting capillary blood for glucose and lipid profile, and non-fasting urinary samples for sodium) to identify raised blood glucose, raised blood cholesterol, and sodium intake. Appointments were given on consent with the respondents for collection of biochemical markers. Trained nurses collected the blood samples using fingertip puncture which were analysed using a standard portable dry chemistry device. Calibrated and validated measuring instruments were used throughout the survey for blood parameters (CardioChek^®^ Plus analyser) as provided by WHO. Spot urine samples were collected from the participants who were asked to collect a sample in the evening before fasting, which was brought to the appointment for blood testing the next morning. Sodium levels in the urine samples were determined using an ion-selective electrode with Abbott Architect c8000 & Roche Cobas 6000 analysers [12]. Quality control measures were taken by each of the health centre laboratories’ analysers to ensure accurate results.

### Data management and statistical analysis

A pilot study of 100 households was conducted in one wilayat (province) with the supervision of the central survey team prior to field work initiation. Participant data was entered by data collectors electronically on a hand-held device programmed with e-STEPS software. For each participant, a unique seven-digit participant identifier code was generated. The data obtained from the laboratory results was combined with the main data for the participant using this unique code. Collected data was downloaded from a central database server following which data management included continuously monitoring data collection, uploading and consolidating processes in the field, validating quality of the data, creating weights, removing duplicate records, and checking inconsistencies namely “jump” errors/outliers, absence of data, excess data, and invalid data. A strict data quality control policy was followed to ensure data reliability. The cleaned data sets were thus ensured for data quality and internal consistency. As part of quality assurance and to increase reliability, a re-interview by telephone was done among 500 random households across all 11 governorates for data validation.

Since the multistage cluster sampling design was adapted with nationwide generalization of findings, the population proportional weight was adjusted for this complex survey design. The sample weight comprised of the inverse probability of selection. The household weights took into account the selection probability of the clusters within each stratum and the size (the number of households) of the cluster. The design weight was adjusted for non-response at the household level. Similarly, the individual weight was adjusted for non-response. Means, medians, proportions, standard errors, and 95% confidence intervals (95% CI) values were calculated to estimate central and dispersion measures and used to assess prevalence differences of NCD risk factors. The independent variables were categorised into demographic variables (age, sex, nationality, education level, employment status, and governorates/regions), behavioural variables (smoking, alcohol consumption, unhealthy diet, and physical activity) and biophysiological parameters (height, weight, blood pressure, blood glucose, total cholesterol). Insufficient fruit/vegetable intake was defined as less than 5 servings of fruit and /or vegetables on average per day as recommended by WHO. Insufficient physical activity was defined as less than 150 minutes of moderate-intensity activity or 75 minutes of vigorous-intensity activity or an equivalent combination of moderate- and vigorous-intensity activity. Overweight was defined as body mass index (BMI) equal or above 25 kg/m^2^, while obesity was defined as those with BMI equal or above 30 kg/m^2^. The cut-off points as recommended by WHO were used in the determination of raised blood pressure (systolic blood pressure >140 mmHg and diastolic blood pressure >90 mmHg) and abnormal biochemical blood and urine samples evaluation (≥6.1 mmol/L & ≥7.0 mmol/L for impaired and raised fasting blood glucose respectively, ≥5.0 mmol/L for raised total cholesterol, and >5 g/day for urine sodium excretion). Statistical procedures for data calculation and analyses were performed through two programs: EpiInfo in collaboration with WHO, and IBM SPSS (Version 20). All the figures and indicators in the tables were calculated using SPSS complex samples analysis.

### Ethical considerations

The survey was approved by the Central Research and Ethical Review & Approval Committee of the Ministry of Health, Sultanate of Oman. (Approval No: 26/2015). Written informed consent was obtained separately during health history collection and measurement of bio physiologic parameters. The confidentiality of the data gathered was maintained. Any waste generated during the biochemical markers’ field procedures was properly safely disposed of as per the recommended protocol. All blood samples were discarded after completing biochemical measurements.

## Results

### Population demographics and socio-economic characteristics

Of the 9053 eligible persons targeted (823 from each of 11 governorates), 6582 persons who were 18 years and above consented to participate in the STEPS survey. The response rate for Step 1 & 2 was 97.9%, and Step 3 which blood was obtained was 93.3% while spot urine collection response rate was 87.2%. Table 1 represents the socio-demographic variables of the sample involved in the survey. The sample population was almost equal between men and women (51% males). A majority of the sample were between the ages of 25 and 44 years (60%) with a mean age of 36.10 ± 13.81. In terms of education level, the largest proportion (34%) had completed secondary school while 23% had completed a University degree with around 27% not having any formal education. 45% of the sample population were not currently working while 22% were working as a government employee. The socio-demographic profile and distribution of samples stratified by sex are also shown in Table 1.

**Table 1:** Socio-demographic profile of the study population stratified by sex.

Variable		Sex					
		Men		Women		Both	
		Count (n)	Percentage (%)	Count (n)	Percentage (%)	Count (n)	Percentage (%)
**Age groups (years)**	18–24 years	344	10.2	374	11.6	718	10.9
	25–34 years	1082	32.2	973	30.2	2055	31.2
	35–44 years	990	29.4	899	27.9	1889	28.7
	45–54 years	527	15.7	454	14.1	981	14.9
	55–64 years	243	7.2	300	9.3	543	8.2
	65 years and above	179	5.3	217	6.7	396	6.0
**Nationality**	Omani	1780	52.9	2865	89.1	4645	70.6
	Non-Omani	1585	47.1	352	10.9	1937	29.4
**Education level**	No formal education	752	22.4	1020	31.7	1772	26.9
	Preparatory or less	654	19.5	334	10.4	988	15.0
	Secondary completed	1116	33.2	1142	35.5	2258	34.3
	University and above	838	24.9	720	22.4	1558	23.7
**Employment status**	Government employee	913	27.1	374	11.6	1287	19.6
	Non-government employee	1637	48.7	129	4.0	1766	26.8
	Self-employed	231	6.9	20	0.6	251	3.8
	Not currently working	583	17.3	2692	83.7	3275	49.8
**Governorates**	Muscat	378	11.2	191	5.9	569	8.6
	Dhofar	377	11.2	310	9.6	687	10.4
	Ad Dakhiliyah	263	7.8	400	12.4	663	10.1
	North Sharqiyah	132	3.9	414	12.9	546	8.3
	South Sharqiyah	138	4.1	394	12.2	532	8.1
	North Batinah	336	10.0	296	9.2	632	9.6
	South Batinah	288	8.6	283	8.8	571	8.7
	Al-Dhahirah	363	10.8	210	6.5	573	8.7
	Al-Buraimi	467	13.9	166	5.2	633	9.6
	Musandam	255	7.6	241	7.5	496	7.5
	Al-Wusta	368	10.9	312	9.7	680	10.3

### Behavioural risk factors

#### Smoking

The overall prevalence of current smokers, including daily and non-daily smokers, was 9% (95% CI: 6.1–11.6) (Table 2). The highest prevalence was observed among men (16%, 95% CI: 11.6–21.3). Omani (6%, 95% CI: 3.6–11.0) smoked less than non-Omani residents (14%, 95% CI: 10.8–18.6). 7% (95% CI: 5.0–10.0) of the population currently smoke tobacco daily. Current daily smoking is most prevalent amongst males (13%, 95% CI: 9.1–19.0) and non-Omani residents (11%, 95% CI: 8.2–15.2).

**Table 2:** Prevalence of behavioural risk factors of non-communicable diseases among the survey participants.

Behavioural Risk Factors	Omani			Non-Omani			Overall		
	Male	Female	Total	Male	Female	Total	Male	Female	Total
**Smoking**									
Current tobacco users (Smoking & Smokeless) (Daily & Non-daily) (%)	14.1 (7.9–23.9)	0.4 (0.1–1.8)	6.3 (3.6–11.0)	18.5 (14.8–23.0)	1.3 (0.7–2.3)	14.2 (10.8–18.6)	15.8 (11.6–21.3)	0.5 (0.1–1.9)	**8.5 (6.1–11.6)**
Current daily tobacco users (Smoking & Smokeless) (%)	12.3 (6.6–21.9)	-	5.6 (3.0–10.0)	14.8 (11.5–18.9)	0.3 (1.0–1.1)	11.2 (8.2–15.2)	13.3 (9.1–19.0)	0.4 (0.1–1.6)	**7.1 (5.0–10.0)**
**Alcohol consumption**									
Alcohol drinkers (drunk alcohol in the past 12 months) (%)	0.9 (0.4–1.9)	-	0.4 (0.2–0.9)	8.6 (3.3–20.4)	6.3 (2.9–13.2)	8.0 (3.3–18.2)	3.9 (1.2–11.8)	0.9 (0.2–4.0)	**2.4 (0.7–8.4)**
**Unhealthy Diet**									
Mean number of servings of fruit/vegetables consumed on average per day (%)	5.0 (4.1–5.9)	5.1 (3.9–6.2)	5.0 (4.6–4.8)	4.1 (3.5–4.7)	4.3 (3.5–4.7)	4.1 (3.6–3.8)	4.0 (3.8–4.1)	4.8 (4.7–4.9)	**4.7 (3.9–5.6)**
Insufficient fruit/vegetable intake (<5 servings of fruit and /or vegetables on average per day) (%)	58.3 (47.0–68.8)	56.9 (45.6–67.8)	57.5 (46.7–67.7)	72.6 (64.8–79.2)	59.5 (53.4–65.4)	69.3 (63.0–75.0)	63.9 (54.6–72.2)	57.3 (47.1–66.9)	**60.7 (51.1–69.5)**
Always or often added salt or salty sauce to their food before or while eating (%)	23.1 (15.1–33.8)	29.3 (20.4–40.1)	26.6 (18.1–37.4)	16.6 (7.4–33.2)	19.7 (12.4–29.9)	17.4 (9.8–28.9)	20.6 (11.8–33.5)	27.9 (19.5–38.3)	**24.1 (15.3–35.9)**
Mean salt intake per day (g)	9.6 (9.5–9.6)	7.4 (7.42–7.48)	8.4 (8.3–8.4)	9.5 (9.50–9.57)	7.5 (7.4–7.5)	9 (8.9–9.0)	9.5 (9.49–9.5)	7.4 (7.39–7.44)	**8.6 (8.596–8.602)**
**Physical activity**									
Median time spent on physical activity on average per day (in minutes)	64	60	60	137	100	120	77	60	**68.6**
Insufficient physical activity (%) (< 150 minutes of moderate-intensity activity/ week, or equivalent)	30.1 (20.6–41.6)	50.5 (37.6–63.4)	41.6 (30.3–53.8)	28.9 (21.7–37.4)	35.7 (16.8–60.5)	30.6 (21.4–41.7)	29.6 (22.0–38.5)	48.5 (34.7–62.6)	**38.6 (27.9–50.6)**

#### Alcohol consumption

Alcohol consumption was observed in 2% (95% CI: 0.7–8.4) of the participants (Table 2). The prevalence of alcohol consumption was higher in males (4%, 95% CI: 1.2–11.8) compared to females (0.6%, 95% CI: 10.2–4.0), and higher among non-Omani residents (8%, 95% CI: 3.3–18.2) than Omani nationals (0.4%, 95% CI: 1.2–11.8).

#### Unhealthy diet

Overall, the mean number of servings of fruit or vegetables consumed per day on average was 4.8 with higher means observed in the overall female (5.0) and Omani (5.0) population (Table 2). A large proportion (61%, 95% CI: 51.1–69.5) of the population reported eating less than the WHO-recommended 5 servings of fruit or vegetables per day. The prevalence of insufficient fruit or vegetable intake was higher in men (64%, 95% CI: 54.6–72.2) as compared to female (57%, 95% CI: 47.1–66.9). A lower prevalence was seen among the Omani population (56%, 95% CI: 46.7–67.7) than the non-Omani resident population (69%, 95% CI: 63.0–75.0). 24% (95% CI: 15.3–35.9) of the population also reported adding salt or salty sauce always or often to their food before or while eating, with a higher prevalence among the female (28%, 95% CI: 19.5–38.3) and Omani (27%, 95% CI: 18.1–37.4) population. The mean salt intake per day was found to 8.6g among the survey participants. The mean salt intake per day was found to be higher in males (9.5g) than females (7.4g), and lower among Omani (8g) than non-Omani residents (9g).

#### Physical inactivity

Insufficient physical activity was prevalent among 39% (95% CI: 27.9–50.6) of the respondents (Table 2). Almost half of the women (49%, 95% CI: 34.7–62.6) along with 30% (95% CI: 22.0–38.5) of men were found to have insufficient physical activity. Omani (42%, 95% CI: 30.3–53.8) had a higher prevalence of insufficient physical activity than non-Omani residents (31%, 95% CI: 21.4–41.7). Omani women (51%, 95% CI: 37.6–63.4) were found to have a higher prevalence of insufficient physical activity compared to Omani men (30%, 95% CI: 20.6–41.6). Overall, the median time spent on physical activity on average per day was 69 minutes, with females (60 minutes) and Omani (60 minutes) reporting lower median times than males (77 minutes) and non-Omani residents (120 minutes) respectively.

### Biological risk factors

#### Overweight and obesity

With an overall mean Body Mass Index (BMI) of 28, Almost two thirds (66%, 95% CI: 62.4–69.6) of the population were overweight or obese (body mass index ≥ 25 kg/m^2^) (Table 3). A higher prevalence of overweight and obesity was observed among women (69%, 95% CI: 65.4–73.1) as compared to men (63%, 95% CI: 57.6–88.8). The Omani population (67%, 95% CI: 63.3–69.6) had a slightly higher prevalence of overweight and obesity than the non-Omani resident population (65%, 95% CI: 58.5–71.1). Likewise, obesity (body mass index ≥ 30 kg/m^2^) was more prevalent in females (39%, 95% CI: 34.2–44.7) than males (23%, 95% CI: 19.3–27.7) contributing to an overall prevalence of 31% (95% CI: 26.0–35.7). Omani women (41%, 95% CI: 35.5–46.6) were found to have a higher prevalence of obesity compared to Omani men (28%, 95% CI: 25.2–31.5). The prevalence was higher among Omani (35%, 95% CI: 31.4–39.2) than non-Omani residents (19%, 95% CI: 14.0–25.2). The BMI mean level was also found to be increasing with age among Omani men and women & non-Omani residents’ men for ages up to 64 years (Table 4).

**Table 3:** Prevalence of biological risk factors of non-communicable diseases among the survey participants.

Biological Risk Factors	Omani			Non-Omani			Overall		
	Male	Female	Total	Male	Female	Total	Male	Female	Total
**Overweight and obesity**									
Mean body mass index–BMI (kg/m^2^)	27.7 (26.8–28.6)	29.1 (28.2–29.9)	28.5 (28.49–28.51)	26.4 (25.8–27.9)	28.1 (27.3–28.9)	26.8 (26.79–26.81)	27.2 (27.18–27.21)	28.9 (28.88–28.92)	**28 (27.5–28.5)**
Overweight (BMI ≥ 25) (%)	63.6 (58.5–68.4)	69.0 (64.8–72.9)	66.5 (63.3–69.6)	63.1 (54.5–70.8)	71.5 (65.8–76.5)	65.1 (58.5–71.1)	63.4 (57.6–68.8)	69.4 (65.4–73.1)	**66.1 (62.4–69.6)**
Obese (BMI ≥ 30) (%)	28.2 (25.2–31.5)	40.9 (35.5–46.6)	35.2 (31.4–39.2)	15.5 (10.6–22.1)	29.8 (23.8–36.6)	19 (14.0–25.2)	23.2 (19.3–27.7)	39.3 (34.2–44.7)	**30.7 (26.0–35.7)**
**Raised blood pressure**									
Mean systolic blood pressure (mmHg)	132.5 (132–132.9)	122.2 (121.7–122.6)	126.7 (126.4–127.1)	132.8 (132.2–133.3)	122.6 (121.2–124)	130.2 (129.7–130.8)	132.6 (132.2–132.9)	122.2 (121.8–122.7)	**127.7 (127.4–128)**
Mean diastolic blood pressure (mmHg)	82.1 (81.7–82.4)	78.9 (78.7–79.2)	80.3 (80.1–80.5)	85.3 (85–85.6)	80.5 (79.8–81.2)	84.1 (83.8–84.4)	83.3 (83.1–83.6)	79.1 (78.9–79.4)	**81.3 (81.2–81.5)**
Prevalence of raised blood pressure (%) (≥ 140/90 mmHg)	37.9 (29.5–47.1)	27.8 (24.5–31.3)	32.2 (26.8–38.0)	40.6 (35.4–46.1)	24.0 (19.0–30.0)	36.5 (32.2–41.1)	39.0 (32.0–46.4)	27.2 (24.3–30.4)	**33.3 (28.0–39.1)**
**Raised blood glucose**									
Mean fasting blood glucose (mmol/L)	5.64 (5.57–5.7)	5.83 (5.79–5.88)	5.74 (5.71–5.78)	5.98 (5.88–6.07)	6.19 (6.03–6.34)	6.03 (5.95–6.11)	5.77 (5.72–5.82)	5.88 (5.84–5.93)	**5.82 (5.79–5.86)**
Prevalence of raised fasting blood glucose (%) (≥ 7.0 mmol/L)	13.5 (12.2–14.8)	15.4 (14.5–16.3)	14.5 (13.8–15.3)	18.5 (17.3–19.7)	19.9 (17.7–22.4)	18.8 (17.8–19.9)	15.4 (14.5–16.3)	16.1 (15.2–16.9)	**15.7 (15.1–16.3)**
Prevalence of impaired fasting glycaemia (%) (≥ 6.1 to < 7.0 mmol/L)	10.2 (9.5–10.9)	12.6 (11.9–13.4)	11.5 (11.0–12.1)	12.5 (12.1–12.9)	12.4 (11.6–13.2)	12.5 (11.7–13.3)	11.1 (10.5–11.6)	12.6 (11.9–13.3)	**11.8 (11.4–12.2)**
**Raised blood total cholesterol**									
Mean total cholesterol (mmol/l)	4.61 (4.57–4.65)	4.80 (4.78–4.83)	4.72 (4.69–4.74)	4.54 (4.5–4.58)	4.83 (4.76–4.9)	4.61 (4.57–4.64)	4.58 (4.55–4.61)	4.81 (4.78–4.83)	**4.69 (4.67–4.71)**
Prevalence of raised total cholesterol (%) (≥5.0 mmol/l)	33.3 (31.8–34.9)	39.9 (38.6–41.6)	37.0 (36.0–38.0)	29.6 (28.1–31.2)	36.8 (34.2–39.5)	31.4 (30.1–32.8)	31.9 (30.8–33.0)	39.5 (38.3–40.7)	**35.5 (34.7–36.3)**

**Table 4:** Risk factor estimates of non-communicable diseases by nationality, sex, and age group.

	BMI^†^ Mean (95% CI)	SBP^∫^ Mean (95% CI)	DBP^Δ^ Mean (95% CI)	BG^∏^ Mean (95% CI)	TC^≡^ Mean (95% CI)
**Omani**					
**Men**					
18–24 years	26.0 (23.6–28.4)	128.9 (127.9–129.8)	78.1 (77.1–79.1)	5.1 (5.10–5.18)	4.0 (3.95–4.05)
25–34 years	26.5 (25.6–27.4)	128.5 (127.8–129.3)	80.5 (79.8–81.2)	5.2 (5.17–5.31)	4.5 (4.45–4.55)
35–44 years	29.0 (28.0–30.0)	130.5 (129.9–131.2)	83.9 (83.4–84.3)	5.5 (5.41–5.53)	4.9 (4.87–4.99)
45–54 years	28.5 (27.6–29.5)	137.3 (136.0–138.6)	85.9 (85.2–86.5)	6.5 (6.18–6.85)	4.8 (4.67–4.87)
55–64 years	31.8 (27.4–36.2)	144.0 (142.1–146.0)	86.5 (85.9–88.3)	6.4 (6.21–6.61)	5.3 (5.07–5.62)
65 years & above	26.8 (24.8–28.8)	140.2 (138.4–142.1)	81.2 (80.3–82.1)	6.5 (6.32–6.65)	4.7 (4.63–4.84)
*p value for trend* *	*<0*.*001*	*<0*.*001*	*<0*.*001*	*<0*.*001*	*<0*.*001*
**Women**					
18–24 years	25.6 (24.6–26.5)	112.8 (111.7–113.8)	74.3 (73.7–74.9)	5.1 (5.05–5.16)	4.5 (4.43–4.54)
25–34 years	27.4 (26.2–28.6)	115.0 (114.5–115.6)	76.6 (76.3–76.9)	5.4 (5.32–5.41)	4.7 (4.64–4.74)
35–44 years	31.3 (30.4–32.1)	121.1 (120.3–121.9)	80.3 (79.9–80.8)	5.8 (5.74–5.86)	4.9 (4.89–4.99)
45–54 years	31.4 (30.5–32.2)	130.1 (128.7–131.5)	83.3 (82.4–84.2)	6.3 (6.20–6.41)	4.9 (4.87–5.01)
55–64 years	30.2 (28.0–32.5)	139.0 (137.6–140.3)	83.5 (82.8–84.1)	6.6 (6.41–6.75)	5.3 (5.12–5.43)
65 years & above	29.6 (27.4–31.9)	141.9 (140–143.7)	81.2 (79.9–82.5)	7.8 (7.43–8.08)	4.8 (4.69–4.86)
*p value for trend* *	*<0*.*001*	*<0*.*001*	*<0*.*001*	*<0*.*001*	*<0*.*001*
**Non-Omani**					
**Men**					
18–24 years	23.6 (22.3–24.8)	125.1 (123.9–126.3)	77.9 (77.0–78.8)	4.3 (5.20–5.41)	4.1 (4.01–4.18)
25–34 years	26.1 (25.3–26.8)	129.1 (128.1–130.1)	84.1 (83.6–84.5)	5.4 (5.30–5.51)	4.3 (4.19–4.35)
35–44 years	26.5 (25.6–27.4)	133.1 (132.2–133.9)	85.6 (85.0–86.1)	5.8 (5.74–5.95)	4.7 (4.67–4.75)
45–54 years	27.3 (26.4–28.1)	134.8 (133.8–135.8)	86.7 (86.0–87.5)	6.5 (6.30–6.67)	4.8 (4.77–4.89)
55–64 years	27.6 (27.0–28.2)	148.0 (145.9–150.2)	92.0 (91.0–93.6)	7.4 (6.92–7.90)	4.8 (4.71–4.93)
65 years & above	26.6 (25.5–27.7)	145.1 (142.3–147.9)	87.9 (84.4–91.4)	10.1 (8.4–11.79)	4.2 (4.11–4.31)
*p value for trend* *	*<0*.*001*	*<0*.*001*	*<0*.*001*	*<0*.*001*	*<0*.*001*
**Women**					
18–24 years	26.1 (23.4–28.7)	108.5 (106.3–110.6)	71.6 (69.6–73.5)	5.3 (5.14–5.41)	4.6 (4.41–4.76)
25–34 years	26.5 (25.9–27.2)	113.3 (112.2–114.3)	76.8 (75.8–77.7)	5.4 (5.31–5.49)	4.5 (4.42–4.55)
35–44 years	29.0 (27.9–30.1)	122.4 (120.4–124.5)	81.9 (80.6–83.2)	5.8 (5.69–5.96)	5.3 (5.13–5.47)
45–54 years	30.4 (29.2–31.6)	136.4 (132.2–141.4)	87.0 (84.9–89.2)	7.6 (7.12–8.16)	4.5 (4.39–4.65)
55–64 years	28.8 (27.3–30.5)	140.3 (133.1–147.6)	84.4 (81.8–87.0)	7.6 (7.10–8.14)	5.3 (5.06–5.59)
65 years & above	28.5 (27.9–29.0)	162.6 (159.9–165.2)	93.3 (91.6–95.1)	11.6 (8.83–14.32)	5.0 (4.66–5.26)
*p value for trend* *	*<0*.*001*	*<0*.*001*	*<0*.*001*	*<0*.*001*	*0*.*016*

^†^Body Mass Index;

^**∫**^Systolic Blood Pressure;

^**Δ**^Diastolic Blood Pressure;

^**∏**^Blood Glucose;

^**≡**^Total Cholesterol;

*p-value for trend adjusted for age

#### Raised blood pressure

The prevalence of raised blood pressure, including those on medication for raised blood pressure, was 33% (95% CI: 28.0–39.1) (Table 3). A higher prevalence was observed among men (39%, 95% CI: 32.0–46.4) than women (27%, 95% CI: 24.3–30.4). Omani (32%, 95% CI: 26.8–38.0) had a lower prevalence of raised blood pressure compared to non-Omani residents (37%, 95% CI: 32.2–41.1). The prevalence of raised blood pressure was found tending to significance (p = .058) by sex and nationality. The mean systolic and diastolic blood pressure was found to be gradually increasing among Omani & non-Omani residents’ men and women up to 64 years of age (Table 4).

#### Blood glucose

Of the survey participants, 16% (95% CI: 15.1–16.3) were found to have raised blood glucose (Table 3). The prevalence was slightly higher among women (16%, 95% CI: 15.2–16.9) than men (15%, 95% CI: 14.5–16.3). A lower prevalence of raised blood glucose was seen in Omani (15%, 95% CI: 13.8–15.3) as compared to non-Omani residents (19%, 95% CI: 17.8–19.9). Impaired glycaemia was prevalent among 12% (95% CI: 11.1–12.2) of the population. Higher prevalences were observed among women (12.6%, 95% CI: 11.9–13.3) and non-Omani residents (13%, 95% CI: 11.7–13.3) as compared to men (11%, 95% CI: 10.5–11.6) and Omani (12%, 95% CI: 11.0–12.1), respectively. The mean blood glucose level estimate is gradually increasing with age among Omani & non-Omani residents’ men and women (Table 4).

#### Raised total cholesterol

The overall prevalence of raised total cholesterol in the population was 36% (95% CI: 34.7–36.3) (Table 3). The prevalence was higher among women (40%, 95% CI: 38.3–40.7) than men (32%, 95% CI: 30.8–33.0). Higher prevalence was also observed in Omani (37%, 95% CI: 36.0–38.0) than non-Omani residents (31%, 95% CI: 30.1–32.8) population. The total cholesterol mean level was also increasing with age among Omani men and women and non-Omani residents’ men and women up to 64 years of age (Table 4).

### Combined risk factors

Combined risk factor analysis was performed with five components: current daily smokers, consumption of less than 5 servings of fruit and vegetables per day, insufficient physical activity, overweight or obesity, and raised blood pressure or currently on medication for raised blood pressure. The analysis of combined risk factors revealed that 95% of the population had more than one risk factor (Table 5). Overall, it was found that 33% (95% CI: 26.6–40.4) of the population had three or more risk factors for chronic disease.

**Table 5:** Summary of combined risk factors by sex, nationality, and age groups.

Age Category	0 NCD risk factors					3 or more of the NCD risk factors				
	Sex		Nationality		Overall	Sex		Nationality		Overall
	Men	Women	Omani	Non-Omani		Men	Women	Omani	Non-Omani	
**18 years & above** (%)	4.9 (2.5–9.3)	5.4 (2.8–10.2)	5.5 (3.1–9.5)	4.3 (3.0–6.1)	**5.1** (3.2–8.1)	35.3 (28.8–42.5)	30.6 (23.0–39.5)	32 (24.3–40.9)	36.1 (32.9–39.5)	**33.2** (26.6–40.4)
18–24 years (%)	10.1 (4.5–21.1)	8.3 (3.7–17.5)	8.8 (4.8–15.7)	13.2 (5.6–28)	**9.3** (5.5–15.1)	19.9 (12.4–30.3)	19.7 (11.5–31.8)	19.2 (13.3–27.0)	25.2 (15.2–38.8)	**19.8** (13.9–27.4)
25–34 years (%)	5.8 (2.5–13.1)	7.0 (3.3–14.0)	7.4 (3.5–15.2)	4.1 (2.4–7.1)	**6.3** (3.2–11.9)	29.3 (22.1–37.8)	20.5 (11.2–34.5)	25.5 (15.8–38.3)	26.0 (22.5–29.9)	**25.7** (18.5–34.4)
35–44 years (%)	3.1 (1.4–6.7)	4.8 (2.3–9.8)	4.1 (2.4–6.8)	3.5 (2.4–5.1)	**3.9** (2.7–5.6)	41.3 (35.0–48.0)	30.7 (24.8–37.4)	37.1 (29.9–45.0)	34.8 (29.6–40.5)	**36.4** (31.0–42.1)
45–54 years (%)	3.9 (2.5–6.2)	1.3 (0.4–4.7)	2.5 (0.8–7.9)	0.5 (0–5.9)	**2.8** (1.8–4.3)	40.6 (32.4–49.3)	39.5 (28.9–51.2)	34.0 (22.9–47.2)	52.4 (45.5–59.1)	**40.1** (31.6–49.3)
55–64 years (%)	1.9 (0.4–8.2)	2.1 (0.5–8.6)	5.2 (1.5–16.4)	-	**2.0** (0.6–7.1)	49.3 (36.2–62.6)	46.7 (31.9–62.1)	46.1 (29.1–64.0)	54.5 (44.0–64.6)	**48.0** (35.1–61.2)
65 years & above (%)	0.9 (0.1–5.9)	7.7 (2.7–19.9)	5.5 (3.1–9.5)	-	**4.7** (1.5–13.8)	50.7 (33.3–67.9)	52.9 (41.4–64.1)	51.4 (38.0–64.5)	56.1 (42.0–69.3)	**51.9** (39.6–64)

## Discussion

This nationally representative STEPS survey is the largest survey conducted in Oman which focused on collecting comprehensive information on both modifiable behavioural risk factors (smoking, alcohol consumption, physical inactivity, and unhealthy diet) and biological risk factors (overweight and obesity, raised blood pressure, raised blood glucose, and raised total cholesterol) for NCDs. The 2008 World Health Survey (WHS) was the last survey done in Oman to assess the national prevalence of some of the risk factors of NCDs [13]. The current survey demonstrated that the Sultanate of Oman has a high prevalence of overweight and obesity, raised blood pressure, raised total cholesterol, insufficient fruit or vegetable intake, salt intake, and insufficient physical activity.

### Behavioural risk factors

In this survey, smoking was found to be mainly prevalent among males and the non-Omani resident population. The overall prevalence of current smoking (9%) is consistent with the 2008 WHS (9%). The prevalence of current smoking among men (16%) has also remained almost constant from 2008 (15%). Even with an increased trend in tobacco smoking attributed to urbanization and underlying cultural factors, the government has taken into account the issue of smoking and has implemented several measures to control the usage among the common public. According to the Royal Decree 43/2018, the advertisement of tobacco products is banned in Oman [14]. There has also been a ban on smoking in public places since 2010 [15]. As a result, Oman also has the lowest prevalence rate in the EMRO region, in contrast with other countries in the region some of which have the highest prevalence rates worldwide [16]. Jawad et al. [16] suggests that Oman needs to adopt policies recommended by WHO’s MPOWER package in order to maintain the current low prevalence of smoking.

Alcohol use was among the least frequent risk factors in the Sultanate of Oman, with the prevalence at just around 2% of the total population. There was a significant difference found between the alcohol use prevalence among Omani nationals and non-Omani residents. The prevalence among Omani was very low owing to cultural factors and strict compliance to religious values. Non-Omani residents were the largest proportion consuming alcohol in Oman at 8%. Table 6 demonstrated that Oman is higher than most other EMRO countries—however when only nationals are taken into account, prevalence rates are in line with other EMRO countries. It is important to note that alcohol availability has also been regulated by stringent laws and monitored by the government sector in Oman [17, 18]. Also, given that alcohol consumption is somehow stigmatized in society, low reported prevalence may also be in part an underestimation, so results should be interpreted with some caution.

**Table 6:** Comparison of risk factors from recent STEPS surveys in various EMRO countries.

	Risk Factor Topic	Oman* (2017)	Egypt (2017)^†^	Qatar (2012)^†^	Iraq (2015)^†^	Lebanon (2017)^†^	Kuwait (2014)^†^
**Behavioural Risk Factors**	Current tobacco smoking (%)	8.0	22.7	16.4	20.7	38	20.5
	Current daily tobacco smoking (%)	6.7	18.4	14.7	19.6	75.6	18
	Alcohol use (in past 30 days) (%)	1.6	0.8	NA	0.6	23.4	0.3
	Insufficient fruit/vegetable consumption (%)	61	90.3	91.1	79.2	73.4	83.8
	Salt intake (g)	8.6	9	NA	NA	NA	NA
	Insufficient physical activity (%)	39	24.9	45.9	47	61	62.6
**Biological Risk Factors**	Overweight & Obesity (%)	66	63	70.1	65.4	62.8	77.2
	Obesity (%)	36	35.7	41.4	33.5	28.6	40.2
	Raised Blood Pressure (%)	33.3	29.5	32.9	35.6	32.8	25.1
	Raised Blood Glucose (%)	15.7	15.5	16.7	13.9	9.4	14.6
	Raised Total Cholesterol (%)	36	19.2	21.9	39.6	48.8	55.9

* Inclusive of non-Omani residents;

^**†**^ Country comparison data obtained from latest country STEPS reports (www.who.int/ncds/surveillance/steps/reports/en/)

Insufficient intake of fruit and vegetables intake per day was found to be fairly high at 61% overall, even though the average consumption of fruits and/or vegetables per day of 4.8 servings seen in the survey is close to the recommendation. Furthermore, Oman is the lowest in terms of insufficient intake of fruits and/or vegetables in recent STEPS surveys conducted in the EMRO region while Qatar and Egypt reported prevalence rates of above 90% (Table 6) [19]. Also on a positive note, the trend of insufficient fruit and vegetables intake decreased from 68% to 61%, and among the Omani population from 70% to 58% in the 2008 WHS as compared to this survey, respectively. There was also a significant difference found among unhealthy diet variables between Omani nationals and non-Omani residents. A serious concern was the mean salt intake per day (9g) which was found to be almost double the recommended amount (5g). This could be attributed to persons adding salt or salty sauce to their food always or often before or while eating which was found to be 24% in this survey. The STEPS survey conducted in Egypt which also performed urine analysis reported the same high level of salt intake [19]. The current survey revealed that women had the highest prevalence of adding salt to the food. Women can have a contributory role in this risk factor as are an important part of the family especially in food preparation. It is also vital that knowledge of healthy eating habits starts young as the prevalence of adequate intake of fruit and vegetables in the eleven EMR countries among adolescents was found to be low [20]. Strategies and/or other alternatives to reduce salt intake, a matter that may require health promotion and education to improve knowledge about sources of salt, should be thought of in order to reduce/modify the risk. The health sector has an important role in determining salt content of processed foods and initiate discussions with the food industry on means to address their reformulation.

In terms of physical activity, WHO recommends exercising at least 150 minutes of moderate or vigorous physical activity weekly. However, it was found in this survey that the median time spent even on total physical activity was only 69 minutes. Omani nationals and women spent only one hour on average for total physical activity per day. This led to 39% of the population having insufficient physical activity, which was consistent with 2008 OWHS (42%) [6]. There was highly significant difference found among Omani nationals and non-Omani residents on the median time spent on physical activity per day. The prevalence of insufficient physical activity in Oman was higher than Egypt (25%) and lower than Qatar (46%), Iraq (47%) Lebanon (61%) and Kuwait (63%) [21]. Modernisation coupled with the hot, humid climate lead to people resorting to using private cars for even fairly short distances thus attributing to the lack of regular outdoor physical activity [22]. However, health promotion, education, transport modality planning, and policy should be brought together to address this multifactorial issue.

### Biological risk factors

A serious cause for concern is the prevalence of overweight and obesity (BMI ≥25) which stands at 66% in Oman. There has been a dramatic increase in the prevalence of overweight and obesity among the Omani population from 54% in 2008 WHS to 67% in 2017 [6]. Also alarming is the prevalence of obesity (BMI ≥30) among the Omani population which is at 35% overall (up from 24% in 2008 WHS) and strikingly among women at 41% (up from 24% in 2008 WHS). The survey findings are Gulf Cooperation Council (GCC) in line with the prevalence of overweight and obesity in the as well as WHO EMRO countries [23, 24]. This remarkable change is due to the changes of lifestyle and increase in socioeconomic status. With urbanisation, the availability of junk and fast food has increased, and hence increased public awareness of balanced healthy eating habits are imperative to mitigate this availability.

The prevalence of raised blood pressure from this survey was reported as 33%, which means that one in three people in the population had high blood pressure, a prevalence level that calls for attention from policy makers, health professionals, and civil society to address this multifactorial problem. Also, the prevalence of raised blood pressure among men is around 10% higher than women, so specific targeted interventions to this group are vital to halt the rise and reduce the prevalence of raised blood pressure overall. Interestingly, the same phenomenon was reported by WHO among the EMRO region as well [25]. In terms of the Omani population, the trend of raised blood pressure seems to be reduced from 40% in 2008 to 32% in this survey. Several researchers consistently report an increasing trend in raised blood pressure in the region [26, 27]. The systolic and diastolic mean among the survey population was found to be close to the normal range. However, as expected in terms of age, there is an increasing trend (*p<0*.*001*) of systolic and diastolic blood pressure up to 64 years of age. In 2015, the Oman Heart Association (OHA) released the internationally recommended guidelines in management of hypertension to be followed [28]. In addition, the national screening program for 40 years and above may have also had an influence to help to reduce the prevalence of increased blood pressure.

The observed prevalence of raised blood glucose in Oman is currently at 16% showing a steady increase. The prevalence trend in raised blood glucose among the Omani population is also on the rise from 12% in 2008 to 15% in 2017. The prevalence of raised blood glucose was similar to Egypt (16%), lower than Qatar (17%), but higher than Kuwait (15%), and Iraq (14%) [21]. Similarly, the prevalence of impaired fasting glycaemia stands at 12%, a three-fold increase from 2008 WHS (4%). This pre-diabetic group is a significant group, which should be targeted in order to treat them early, hence providing an opportunity to reduce health impacts and costs of diabetes treatment later on in the progression of the disease. Our study found a highly significant difference between Omani nationals and non-Omani residents in terms of prevalence of raised and impaired fasting blood glucose. With age, a significant increase was also found in the mean blood glucose level among Omani and non-Omani residents in our study.

The national prevalence rate of raised total cholesterol was reported as 36% in the survey, which was in line with the global prevalence of raised total cholesterol [29]. Our results are lower than a majority of Gulf countries where the prevalence was 50% or higher [30]. The prevalence among women was higher than men, which also seemed to be the trend globally [29]. The prevalence among the Omani population was observed at 37%, which was higher than the prevalence reported in 2008 (34%) [6]. There was also a significant difference found between Omani nationals and non-Omani residents. The reported overall total cholesterol level is lower than Kuwait (56%) and Iraq (40%) but higher than Egypt (19%), Qatar (22%) [21]. It is important for the public to recognise that keeping cholesterol levels in check is vital for their overall cardiovascular health.

### Number of behavioural or biological risk factors

Only 5% of the study population were found to be free of all the five NCD risk factors. Furthermore, the overall proportion of adults in Oman with three or more risk factors was 33%, with the proportion substantially increasing to almost 45% among the 45 years and above age group. There were no significant differences found between Omani nationals and non-Omani residents. Among those 45 years and above, the overall prevalence of three or more risk factors was found to be 45% with men and women in this category having equal prevalence (both 45%) of three or more risk factors. The overall prevalence of those having three or more risk factors increased dramatically from around 20% among 18–24 years to 52% among 65 years and above. This analysis of combined risk factors is an alarming figure which informs us that the burden of NCDs is likely to increase in the future if no prompt action is taken by the stakeholders involved.

### Strengths and limitations

There are several strengths to add. Firstly, this the largest study comprehensively collecting data on NCD risk factors surveying 9053 eligible persons. The survey was conducted on a national level, and hence the findings are very well representative for implications at national and regional level. The survey was also met with a high response rate which ensures the results are representative of the target population. Validated and piloted instruments were used during the survey, and the data collectors and their supervisors were provided with extensive training. A gold standard 24-hour urine subsample collection was also done along with spot urine sample to assess salt, creatinine, and potassium intake. Data monitoring was done continuously by a central team to ensure reliability of uploaded data.

An important limitation of this study is the potential bias on self-reporting tobacco and alcohol use due to social customs, adherence and practice of the population to Islamic values, and stigmatization. Hence, the prevalence of smoking and alcohol consumption may be underestimated. Furthermore, medical history was self-reported and not cross-verified with existing health records. Lastly, our study was a cross-sectional design with its own inherent limitations regarding causal inferences.

### Survey implications

The ongoing programs for NCDs in Oman need be optimally harmonized to address the challenges of the rising prevalence of NCD risk factors from the 2008 World Health Survey. The most critical which need to evaluated are national multi-stakeholder dialogue and accountability. Furthermore, implementing appropriate policies and regulations in tandem with engaging the public is imperative to combat these NCD risk factors. Identifying, prioritising, and scaling up of cost-effective lifesaving interventions along with WHO NCD ‘best-buys’ are recommended to be included in these programs to protect people through ‘saving lives, spending less’ [31]. Of great consequence, is to ultimately create healthy cities and environments which would lead to halt and reduction of these increasing risk factors.

## Conclusions

The Oman National Non-communicable Diseases Risk Factors Survey (STEPS) provides essential information on key NCD indicators by age group, sex, and governorates. The study reveals that that around two-third of the population are either obese or overweight and 61% have insufficient fruit and vegetable intake with almost 40% having insufficient physical activity. In addition, it showed that almost one third have raised blood pressure and blood cholesterol and 16% have raised blood glucose. The prevalence of multiple risk factors determined that 95% of the population were found to have more than one risk factor. Three or more risk factors were found among 33% of population aged 18 years and above and 45% of the population aged 45 years and above.

NCDs are currently one of the top priorities globally as well as for the Omani Ministry of Health with Oman chosen by WHO as one of 11 countries to be monitored for combating NCDs and their risk factors. There is a need to prioritize NCD prevention and control at both the national and governorate level with the multisector governmental as well as societal support as it is an emerging threat to health, social and economic development. The strengthening of implementation of Oman’s national policy for diet, physical activity and health along with ensuring continuous engagement with the agricultural sector will promote healthy diet among the population. In order to prevent NCDs, we must build awareness of NCD risk, educate people, address the social determinants of health, discourage tobacco use and excessive alcohol intake. Regarding physical activity, creating conditions or spaces that are suitable for physical activity, raising population awareness about needed effort and benefits, and promoting more physical activity at all possible times and areas, including work and among the youth, are potential areas for intervention and health improvement. Introduction of legislations & guidelines on production, packaging and responsible marketing of food will aid in reducing consumption of unhealthy foods. Most importantly, the integration of these key NCD indicators in national health surveys will enhance existing data in order to achieve proper planning and future projections for NCD prevention and control.

## Supporting information

S1 Appendix Survey sample size calculation. (DOCX)
