# Supplementary material for: Prevalence of risk factors of non-communicable diseases in the Sultanate of Oman: STEPS survey 2017
Source: PLoS One. 2021 Oct 28;16(10):e0259239. doi: 10.1371/journal.pone.0259239 (PMC8553065; doi:10.1371/journal.pone.0259239)
Supplement: S1 Appendix — (DOCX) [file pone.0259239.s001.docx]

### **S1 Appendix. Sample Size Calculation**

A WHO STEPS standard formula was used in the calculation of the sample size based on the guidelines/recommendations of the STEPS survey.

n= Z^2^ * P (1-P)/d^2^

Where;

n= the required sample size

Z= the probability value associated with the confidence level

P= the prevalence rate of NCDs risk factors in the country

d= the desired margin of error (precision).

In turn:

Z= 1.96 (95% confidence interval as recommended)

P= 0.5 (the conservative value of prevalence rate)

e= 0.05 (as recommended in the guidelines)

Using these values, the initial calculation was: n= 384 households

Also taken into account for sample size calculations were: a value of design effect, as recommended in STEPS surveys, to be 1.5; and, an anticipated response rate of 70% was estimated. By adjusting the sample by these factors, the sample size per cluster (governorate) results in:

$$n=\frac{384 x 1.5}{0.7}=823\mathrm{households}$$

To get the desired precision and overall figures adequate for age-sex groups and for overall estimate on governorates level, the sample size was:

n= 823 x 11 = 9053 households, with one individual selected per household

The 9053 households were distributed equally by governorate (823), proportional to nationality, according to the ratio of Omani and non-Omani households in each governorate
